# Melatonin Regulates the Expression of VEGF and HOXA10 in Bovine Endometrial Epithelial Cells through the SIRT1/PI3K/AKT Pathway

**DOI:** 10.3390/ani14192771

**Published:** 2024-09-25

**Authors:** Qi Li, Ying Tang, Yanru Chen, Bo Li, Hongzhan Wang, Shicheng Liu, Samson O. Adeniran, Peng Zheng

**Affiliations:** 1 College of Animal Science and Technology, Northeast Agricultural University, Harbin 150030, China; liqi04030216@163.com (Q.L.); 15905233543@163.com (Y.T.); yanruchen1012@gmail.com (Y.C.); 15161330947@163.com (B.L.); 18937298367@163.com (H.W.); 18903653925@163.com (S.L.); 2 Institute of Animal Science, Chinese Academy of Agricultural Sciences, Beijing 100193, China; 3 Biotechnology Unit, Department of Biological Sciences, College of Basic and Applied Sciences, Mountain Top University, Ibafo 110115, Nigeria; samadeniran328@gmail.com

**Keywords:** endometrial epithelial cells, melatonin, PI3K, VEGF, HOXA10

## Abstract

**Simple Summary:**

The low embryo attachment rate and early embryo loss of dairy cows are important factors restricting the improvement of dairy cow fertility and are also the key scientific problems that need to be solved urgently in dairy cow production and embryo transfer. In this study, we used RT-qPCR, Western blot, immunofluorescence, and so on in cultured bovine endometrial epithelial cells to investigate the mechanism by which melatonin regulates the expression of VEGF and HOXA10 through SIRT1, PI3K, AKT, and miRNA. The results indicate that melatonin regulates the expression of VEGF and HOXA10 through the SIRT1/PI3K/AKT pathway and promotes the establishment of receptivity in bovine endometrial epithelial cells. The results indicate that melatonin negatively regulates the expression of bta-miR-497 and bta-27a-3p in bovine endometrial epithelial cells through the SIRT1/PI3K/AKT pathway and positively regulates the expression of VEGF and HOXA10. In this regulatory network, bta-miR-27a-3p negatively modulates HOXA10 expression, while bta-miR-497 negatively modulates VEGF expression.

**Abstract:**

Melatonin plays a critical role in regulating embryo attachment in ruminants. While numerous studies have investigated its effects on early embryo development in vitro, the precise mechanisms by which melatonin influences the receptivity of endometrial epithelial cells in dairy cows remain unclear. The prerequisite for embryo implantation is the specific physiological condition of the endometrium that allows the embryo to implant, also known as endometrial receptivity. In addition to this, endometrial cells undergo processes such as proliferation, differentiation, and renewal, which makes the embryo more easily implanted. In this study, bovine endometrial epithelial cells were cultured and treated with melatonin, Silent Information Regulator 1 (SIRT1) inhibitor (EX527), and protein kinase B (AKT) phosphorylation inhibitor (periposine). RT-qPCR, Western blot, and immunofluorescence analysis were performed to investigate the effects of melatonin on the expression of target gene (SIRT1); cell proliferative genes, phosphatidylinositol-4,5-bisphosphate 3-Kinase (PI3K), AKT, cyclinD1, cyclinE1; and receptive genes (Leukemia Inhibitory Factor (LIF), Vascular Endothelial Growth Factor (VEGF), Homeobox Structure Gene 10 (HOXA10)). Additionally, microRNA (miRNA) mimics and inhibitors were used to transfect the cells to study the regulatory relationship between miRNA and receptive genes. Results indicated that melatonin activates the PI3K/AKT signaling pathway, upregulates cyclinD1 and cyclinE1, and promotes the proliferation of bovine endometrial epithelial cells. Melatonin also upregulated the expression of VEGF and HOXA10 and downregulated the expression of bta-miR-497 and bta-miR-27a-3p through SIRT1/PI3K/AKT signaling pathway. Further, bta-miR-497 and bta-miR-27a-3p were found to negatively regulate VEGF and HOXA10, respectively. Therefore, melatonin regulates the expression of VEGF and HOXA10 through the SIRT1/PI3K/AKT pathway and promotes the establishment of receptivity in bovine endometrial epithelial cells.

## 1. Introduction

Melatonin, an indole hormone mainly synthesized by the pineal gland, plays a vital role in regulating circadian and seasonal rhythms, as well as seasonal reproductive changes in animals [1]. It has significant implications for animal reproduction. Studies on male animals have demonstrated that melatonin influences spermatogenic cell morphology in Suffolk sheep and modulates cAMP synthesis in testicular cells during in vitro experiments [2]. Additionally, in vivo research has shown that melatonin can reverse radiation-induced abnormalities in rat sperm morphology, count, and ultrastructure [3]. In female animals, melatonin impacts the morphology of luteal and follicular granulosa cells, and it also regulates endometrial vascular permeability and shedding [4]. Furthermore, melatonin promotes the development of the corpus luteum and enhances progesterone secretion [5]. In dairy cows, melatonin combined with exogenous progesterone improves hemodynamics in follicles, corpus luteum, ovaries, and uterus while reducing nitric oxide production, thereby enhancing fertility under heat stress conditions [6].

Embryo implantation is a critical and complex process that requires correct communication between the blastocyst and the uterus. During embryo implantation, the interaction between embryo trophoblast cells and endometrial epithelial cells results in the formation of new tissue, a process often accompanied by neovascularization [7,8]. Vascular Endothelial Growth Factor (VEGF) is a cytokine that induces angiogenesis and regulates vascular permeability [9]. VEGF and its receptors are expressed in reproductive tissues, such as the endometrial vasculature and implantation sites in mice [10]. Additionally, VEGF protein expressed in the blastocyst binds to endometrial receptors to induce angiogenesis at the implantation site, all of which indicate that VEGF plays a critical role during embryo implantation [11]. Homeobox Structure Gene 10 (HOXA10) in the HOX family is a transcription factor containing homeoboxes, and its correct expression can regulate embryonic development and is crucial for establishing endometrial receptivity [12]. HOX is involved in the whole process of embryo implantation in ruminants, but the research on embryo implantation in ruminants, particularly in cow, is not clear [13]. Our previous studies have demonstrated that Leukemia Inhibitory Factor (LIF) regulates the expression of HOXA10 in the bovine endometrium through the STAT3 transcription factor, thereby enhancing receptivity and facilitating successful embryo attachment [14]. Consequently, in subsequent studies on the receptivity of bovine endometrial epithelial cells, we will maintain a focus on the HOXA10 factor.

The establishment of uterine receptivity, embryonic development before implantation and the occurrence of physiological events such as embryo implantation are regulated by cytokines, chemokines, growth factors, and steroids. Evidence suggests that melatonin plays a role in maintaining uterine homeostasis through multiple pathways, thereby improving uterine physiological processes such as implantation and decidualization [15]. Experimental studies in mice have shown that melatonin can increase estradiol levels, which benefits implantation but shortens the uterine receptive period [16]. Additionally, melatonin has been observed to increase the implantation rate and litter size in mice by inducing the expression of MTNR1B (MT2) and arylalkylamine N-acetyltransferase (AANAT) in the uterus during early pregnancy [17]. While the effects of melatonin on the ovary and follicles are well documented, the specific signal transduction pathways involved in endometrial receptivity remain unclear.

Sirtuin1 (SIRT1), a member of the histone deacetylase sirtuin family, plays a crucial role in counteracting aging processes in the reproductive system and facilitating embryo implantation. Notably, the expression levels of sirtuins (SIRT1, SIRT3, and SIRT6) are positively correlated with the follicular reserve, serving as markers of ovarian aging in mice [18]. Deletion of SIRT1 in rats has been shown to lead to the premature depletion of follicles during early developmental stages [19]. Melatonin has emerged as a promising anti-aging agent for treating mice, delaying ovarian senescence through its antioxidant properties, maintaining telomere length, stimulating sirtuin expression, and enhancing ribosomal function [20]. In the context of endometriosis, studies have demonstrated that inhibition of the SIRT1/FOXO3a signaling pathway promotes senescence in endometrial stromal cells (ESCs) and inhibits the ectopic implantation of ESCs [21]. Furthermore, knockout of SIRT1 in normal mice has been associated with reduced male fertility and a significant reduction in embryo implantation sites in females [22]. These findings suggest that melatonin may influence endometrial receptivity, and its underlying mechanisms warrant further investigation.

This study investigates the effects of melatonin on bovine endometrial epithelial cells, focusing on cell proliferation and the expression of receptivity genes HOXA10 and VEGF. Specifically, we explore the regulatory mechanism of the SIRT1/PI3K/AKT signaling pathway on the expression of these genes. The findings aim to enhance our understanding of the interaction between melatonin and the bovine endometrium, providing a theoretical foundation for using melatonin to improve endometrial receptivity and pregnancy rates in dairy cows.

## 2. Materials and Methods

### 2.1. Ethics Statement

The Institutional Animal Care and Use Committee of Northeast Agricultural University, China approved all experimentation protocols (NEAUEC20230248).

### 2.2. Reagents and Chemicals

The bovine endometrial epithelial cell was purchased from Otwo Biotech (Cat. no.: HTX2241, Shenzhen, China). Trizol^®^ (Cat. no.: 15596026, Invitrogen, Shanghai, China), Fetal bovine serum (FBS, Cat. no.: P30-3306, PAN-Biotech, Adenbach, Germany), Reverse transcription kit (Cat. no.: RR047A, Takara, Beijing, China), ROX (Cat. no.: 72986700, Roche, Shanghai, China), BCA kit (Cat. no.: SW101-02, Seven Biotech, Beijing, China), Dimethyl sulfoxide (DMSO, Cat. no.: D2650, Sigma, Shanghai, China), DMEM/F12 (Cat. no.: D8437, Sigma, Shanghai, China), and Lipofectamine^®^3000 (Cat. no.: L3000015, Invitrogen™, Shanghai, China) were used. All the reagents were from Sigma-Aldrich unless otherwise specified.

### 2.3. Cell Culture and Treatment

The experimental material was taken from the laboratory-preserved endometrial epithelial cells of dairy cows. In previous experiments, we identified the purity of the inner membrane cells [23]. And the preserved bovine endometrial epithelial cells have been used as experimental materials to study endometrial receptivity [24,25]. Following previously established methods [24], the bovine endometrial epithelial cells (BEEC) were cultured in DMEM/F12 supplemented with 10% FBS (treated with carbon adsorption to remove endogenous hormones) and 1% penicillin and streptomycin (Cat. no.: SC118-01, Seven Biotech, Beijing, China). The cells were maintained at 37 °C in a humidified atmosphere with 5% CO_2_ in 60 mm Petri dishes, and experiments were initiated when the cells reached 80% confluency. For combined treatments with melatonin and EX527, the cells were divided into four groups: control, 50 nM melatonin, 1.5 μM EX527, and 50 nmol melatonin +1.5 μM EX527. After 24 h of treatment, the cells were harvested for subsequent experiments. Similarly, for combined treatments with melatonin and perifosine, four groups were prepared: control, 50 nM melatonin, 3 μM perifosine, and 50 nM melatonin + 3 μM perifosine. The control group was given nothing but culture medium. After 24 h of treatment, the cells were used for further experimentation. Melatonin, EX527, and perifosine were prepared as concentrated solutions in DMSO and diluted with culture medium to the required concentrations, ensuring the final DMSO concentration was less than 0.1%.

### 2.4. CCK8

Bovine endometrial epithelial cells were seeded in 96-well plates (1 × 10^4^/well) and incubated overnight at 37 °C in 5% CO_2_. Wash the cells inoculated the previous day with PBS (Cat. no.: SC106-01, Seven Biotech, Beijing, China) twice and then change to medium containing MT at a concentration of 50 nM for continued culture for 12 h and 24 h. Another blank well (containing culture medium only) was set up. Discard the old solution, add 90 μL of pure medium and 10 ul of CCK8 solution to each well of the 96-well plate to be tested and incubate at 37 °C for 2 h. A standard enzyme instrument is used to measure the OD value of each experiment hole at 450 nm and detect the change in each cell vitality.

### 2.5. Real-Time Quantitative PCR Analysis

Referring to the previous method [23], the TRIzol reagent (Cat. no.: 15596026, Invitrogen, Shanghai, China) was used to extract total RNA of bovine endometrial epithelial cells. The ultramicrospectrophotometer was used to determine the mRNA concentration. The Takara kit was used for cDNA synthesis. The expression of *PI3K*, *AKT*, *SIRT1*, *VEGF*, *HOXA10*, *LIF*, *cyclinD1*, *cyclinE1*, bta-miR-27a-3p, and bta-miR-497 genes were detected by real-time fluorescence quantitative PCR with cDNAs as template. The RT-qPCR reaction mixture with a total volume of 10 μL contained 1 μL cDNA, 5 μL FastStart Universal SYBR Green Master (ROX), 0.3 μL forward primer (10 μM), 0.3 μL reverse primer (10 μM), and 3.4 μL DEPC-treated water. The PCR program was predenaturated at 95 °C for 30 s. There were 40 cycles of denaturation at 95 °C for 15 s and annealing at 60 °C for 60 s. Each set of experiments was set up in triplicate, and each replicate was run three times. β-actin and U6 genes served as the internal control, and relative mRNAs and miRNAs expression changes were calculated using the 2^-ΔΔCT^ method. All primers were synthesized by BGI Genomics (Beijing, China). Details of the primer sequences are provided in Table 1 and Table 2.

### 2.6. Western Blot Analysis

Protein extraction was performed according to a previously described method [26]. The details of the antibodies used in the present study are shown in Table 3. Proteins were extracted using RIPA lysis buffer (Cat. no.: L3000015, P0013B, Beyotime, Shanghai, China) supplemented with 1 mM PMSF (Cat. no.: L3000015, BL507A, Biosharp, Hefei, China), and protein concentrations were determined using a BCA assay kit. Based on the target protein size, either 10% or 12% sodium dodecyl sulfate-polyacrylamide gel electrophoresis was employed to resolve the protein samples, with 30 μg of protein loaded per well. Following electrophoresis, the separated proteins were transferred onto PVDF membranes (Cat. no.: NW0453, Roche, Shanghai, China), which were subsequently blocked with 5% non-fat milk prepared in TBST (Cat. no.: SW142-01, Seven Biotech, Beijing, China) for 2 h at room temperature. The membranes were then incubated with primary antibodies overnight at 4 °C. After washing with TBST, the membranes were incubated with secondary antibodies for 2 h at room temperature. Finally, protein bands were detected using an Enhanced Chemiluminescence (ECL) solution (Cat. no.: SW133-01, Seven Biotech, Beijing, China) and visualized with an ECL Western blotting detection system (Smartchemi^TM^ image analysis system, Beijing Sage Creation Science Co. Ltd., Beijing, China). Band densitometry analysis was performed using ImageJ software. Each treatment group was analyzed in triplicate.

### 2.7. Immunofluorescence Assay

The BEEC were seeded into a six-well plate and cultured until they reached 80% confluency. The culture medium was then discarded, and the cells were washed with PBS. After removing PBS, 4% paraformaldehyde was added, and the cells were fixed at 4 °C for 10 min. The paraformaldehyde was then removed, and the cells were rewashed with PBS. Following PBS removal, 5% BSA was added to block the cells for 1.5 h at 4 °C. Primary antibodies (SIRT1 rabbit Polyclonal antibody, VEGFA rabbit Polyclonal antibody, Rabbit Anti-HOXA10 Polyclonal Antibody) were applied, and the cells were incubated overnight at 4 °C. The following day, secondary antibodies (Goat Anti-rabbit IgG H&L/FITC) were added and incubated for 2 h at room temperature. The details of the antibodies used in the present study are shown in Table 4. The nuclei were stained with DAPI. Fluorescent images were captured using a confocal microscope (Nikon, Tokyo, Japan). All morphological measurements were conducted in triplicate.

### 2.8. miRNA Mimic, miRNA Inhibitor, and Transfection

The miRNAs binding to VEGF and HOXA10 genes were predicted by TargetScan 8.0 software, and the miRNAs targeting VEGF and HOXA10 genes were selected as miR-497 and miR-27a-3p, respectively. Query the miRBase database https://www.miRBase.org (accessed on 23 October 2023) for mature sequences of miR-497 and miR-27a-3p. MiRNA mimics and miRNA inhibitors (Table 5) were purchased from the HANBIO company (Shanghai, China). The BEEC were transfected with 50 ng of miRNA mimic or 500 ng of miRNA inhibitor using Lipofectamine 3000 (Cat. no.: L3000015, Invitrogen, Shanghai, China) according to the manufacturer’s instructions. Negative control (NC) mimics and NC inhibitors were designed, synthesized, and transfected into cells, respectively. Six hours post-transfection, the transfection medium was replaced with a fresh culture medium, and the cells were incubated for an additional 24 h before proceeding with further experiments.

### 2.9. Statistical Analysis

Data were analyzed using SPSS 20.0 software. When analyzing one variable, between-group comparisons were performed using the T-test, and multi-group comparisons were performed using one-way ANOVA followed by Duncan’s post hoc test. Two-way ANOVA was used to compare three groups when two variables were analyzed. All results are presented as mean ± standard error. In the figures, identical letters and ns indicate no significant difference (*p* > 0.05), whereas different letters and **** denote a significant difference (*p* < 0.05).

## 3. Results

### 3.1. Effects of Melatonin on BEEC Proliferation and Genes Expression

**Figure 1:**
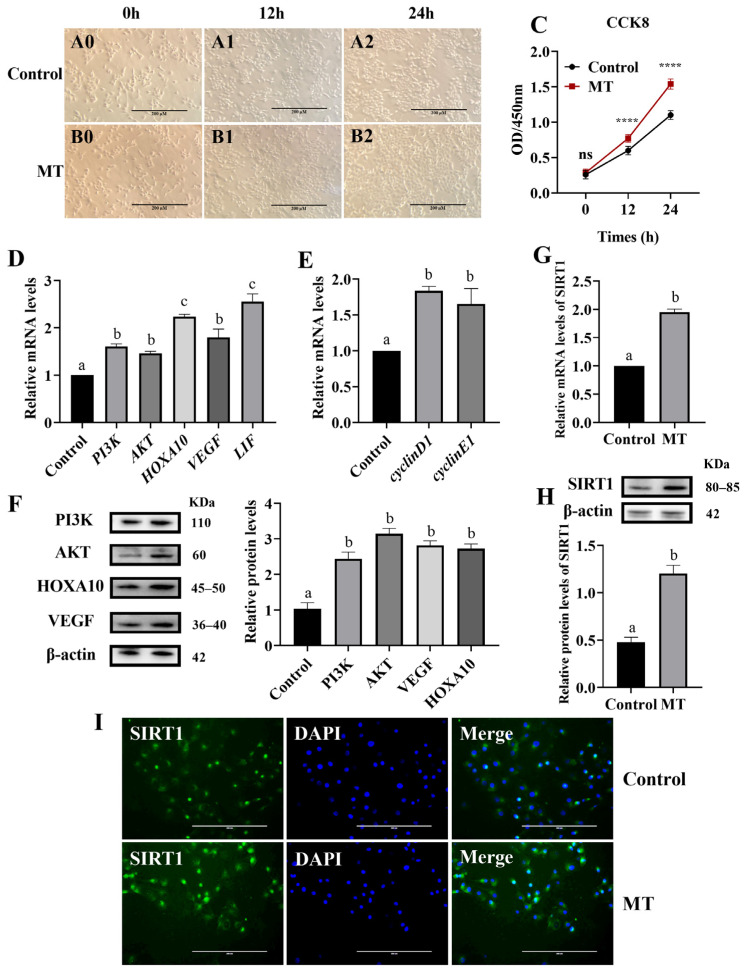
Effects of melatonin on proliferation and gene expression of BEEC (**A0**–**A2**) cell proliferation in the control group (100×); (**B0**–**B2**) cell proliferation in the MT group (100×); (**C**) effect of MT treated bovine endometrial epithelial cells on cell viability at a concentration of 50 nM for 12 h and 24 h; (**D**–**F**) effect of MT at 50 nM on PI3K, AKT, HOXA10, VEGF, LIF, cyclinD1, and cyclinE1 expression in BEEC; (**G**–**I**) effect of MT at 50 nM on SIRT1 expression in BEEC. (**A0**–**A2**,**B0**–**B2**) Bar = 100 μm, (**E**) Bar = 200 μm. MT: Melatonin. The same letters and ns in this Figure mean insignificant difference (*p* > 0.05), while different letters and **** mean significant difference (*p* < 0.05).

Our laboratory previously reported the identification of bovine endometrial epithelial cells [25]. The results of microscopic observation showed that the endometrial epithelial cells of dairy cows were in the shape of “Paving stones”, and the boundary between the cells was clear. According to microscope observation and CCK8, the growth status and speed of cells at 0 h, 12 h, and 24 h were analyzed. Melatonin treatment significantly promoted cell proliferation (Figure 1A0–A2, B0–B2,C). The RT-qPCR analysis showed that melatonin significantly increased the expression of PI3K and AKT genes (*p* < 0.05). Additionally, melatonin significantly increased the expression of cell proliferation-related genes cyclinD1 and cyclinE1, as well as receptivity-related genes (Figure 1D,E) (*p* < 0.05). These findings were corroborated by the Western blot results (Figure 1F) (*p* < 0.05). Furthermore, the RT-qPCR, WB, and immunofluorescence analyses demonstrated that melatonin significantly increased the gene and protein expression of SIRT1 (Figure 1H–I) (*p* < 0.05).

### 3.2. Melatonin Regulates Gene Expression in Cells via SIRT1 

**Figure 2:**
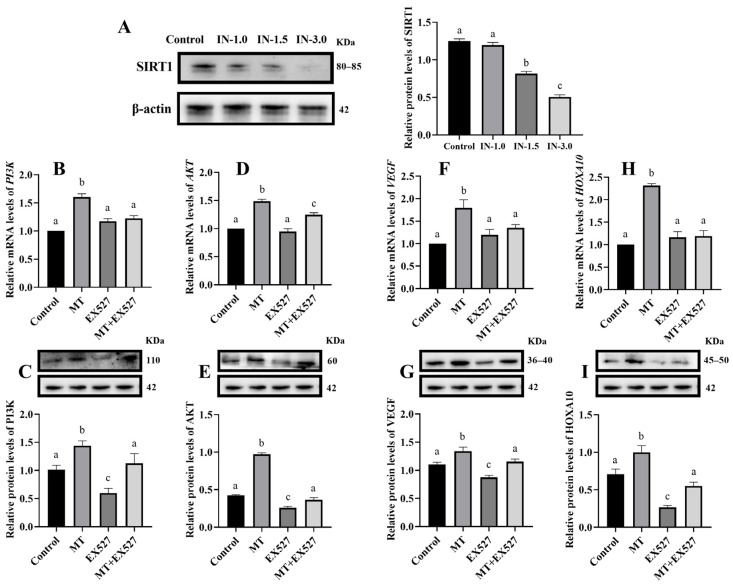
Effect of prevented SIRT1 activation on BEEC gene expression (**A**) SIRT1 protein expression in BEEC treated with different concentrations of EX527; (**B**–**I**) the expression of PI3K, AKT, VEGF, and HOXA10 in BEEC treated with EX527 at 1.5 μM. MT: Melatonin, IN-1.0: 1 μM inhibitor of SIRT1, IN-1.5: 1.5 μM inhibitor of SIRT1, IN-3.0: 3.0 μM inhibitor of SIRT1. The same letters in this Figure mean insignificant difference (*p* > 0.05), while different letters mean significant difference (*p* < 0.05).

The Western blot showed that the expression of SIRT1 protein was significantly decreased in groups treated with 1.5 μM and 3 μM of EX527 (a SIRT1 inhibitor) (Figure 2A) (*p* < 0.05). Consequently, 1.5 μM EX527 was selected as the experimental concentration for SIRT1 inhibition in subsequent studies. RT-qPCR and Western blot analyses demonstrated that compared to the control group, the melatonin (MT) group showed a significant increase in the mRNA and protein expression of PI3K, AKT, VEGF, and HOXA10 (*p* < 0.05). However, in the group co-treated with melatonin and EX527, the expressions of PI3K, AKT, VEGF, and HOXA10 were significantly lower than in the melatonin-only group and showed no significant difference compared to the control group (Figure 2) (*p* < 0.05).

### 3.3. Melatonin Regulates Cellular VEGF and HOXA10 Gene Expression through AKT

**Figure 3:**
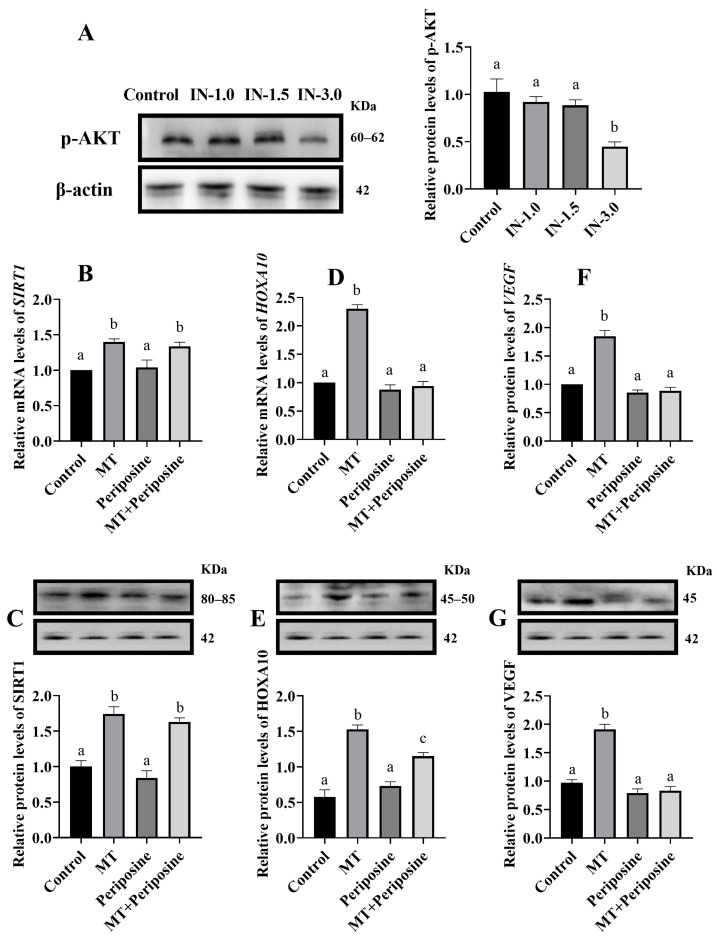
Effect of inhibition of AKT phosphorylation on genes expression in BEEC (**A**) the p-AKT protein expression in BEEC treated with different concentrations of periposine; (**B**–**G**) the expression of SIRT1, HOXA10, and VEGF in BEEC treated with periposine at a concentration of 3 μM. MT: Melatonin, IN-1.0: 1 μM inhibitor of AKT, IN-1.5: 1.5 μM inhibitor of AKT, IN-3.0: 3.0 μM inhibitor of AKT. The same letters in this Figure mean insignificant difference (*p* > 0.05), while different letters mean significant difference (*p* < 0.05).

The Western blot showed that periposine (AKT phosphorylation inhibitor) at a concentration of 3 μM significantly inhibited AKT phosphorylation (Figure 3A) (*p* < 0.05). Consequently, 3 μM periposine was selected for subsequent experiments to inhibit AKT phosphorylation. The RT-qPCR and Western blot indicated that SIRT1 expression in both the melatonin (MT) group and the MT + periposine co-treatment group did not change significantly compared to each other (*p* > 0.05), but both were significantly higher than the control group (*p* < 0.05). This suggests that SIRT1 expression is not affected by AKT phosphorylation (Figure 3B−C) (*p* > 0.05). Furthermore, the expression of VEGF and HOXA10 was significantly higher in the MT group compared to both the control group and the MT + periposine co-treatment group (*p* < 0.05). There was no significant difference in VEGF and HOXA10 expression between the MT + periposine co-treatment group and the control group (*p* > 0.05). This indicates that VEGF and HOXA10 expression is influenced by AKT phosphorylation (Figure 3D−G).

### 3.4. Melatonin Regulates VEGF Expression through miRNA

**Figure 4:**
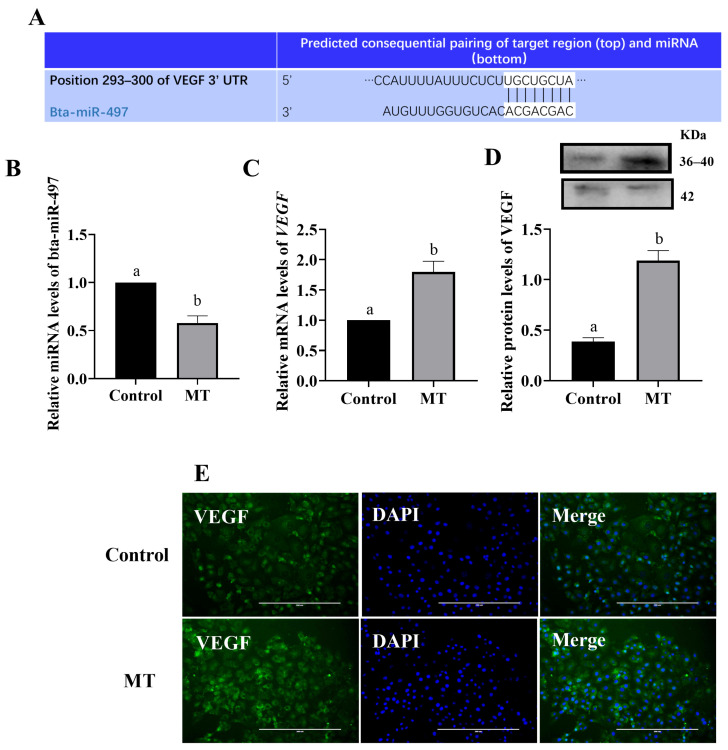
Effect of MT on VEGF and bta-miR-497 expression (**A**) predicted binding sites for bta-miR-497 on VEGF; (**B**) effect of melatonin treatment on the expression of bta-miR-497 in cells; (**C**–**E**) effect of melatonin treatment on the expression of VEGF mRNA and protein in cells. Bar = 200 μm. MT: Melatonin. The same letters in this Figure mean insignificant difference (*p* > 0.05), while different letters mean significant difference (*p* < 0.05).

**Figure 5:**
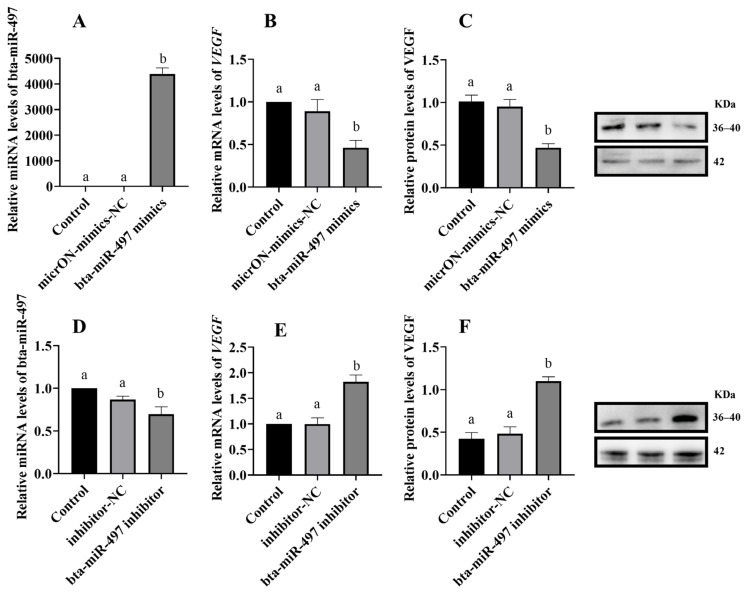
Effect of overexpression and inhibition of bta-miR-497 on the expression of bta-miR-497 and VEGF (**A**) the expression of bta-miR-497 when overexpressing bta-miR-497; (**B**,**C**) the expression of VEGF when overexpressing bta-miR-497; (**D**) the expression of bta-miR-497 during inhibition of bta-miR-497; (**E**,**F**) the expression of VEGF during inhibition of bta-miR-497. NC: negative control. The same letters in this Figure mean insignificant difference (*p* > 0.05), while different letters mean significant difference (*p* < 0.05).

Bioinformatics analysis predicted that miR-497 would bind to the 3′ UTR of VEGF (Figure 4A), potentially regulating VEGF expression. After MT treatment of BEEC, the RT-qPCR results showed a significant decrease in miR-497 expression and a significant increase in VEGF expression (Figure 4B,C) (*p* < 0.05). The Western blot and immunofluorescence analyses further confirmed that VEGF protein expression was significantly elevated (Figure 4D,E) (*p* < 0.05). To validate the regulatory relationship between miR-497 and VEGF, BEEC were subjected to miR-497 overexpression and inhibition experiments. The RT-qPCR and Western blot analyses revealed that overexpression of miR-497 significantly increased miR-497 levels and decreased VEGF gene and protein expression (*p* < 0.05). Conversely, inhibition of miR-497 significantly reduced miR-497 levels and increased VEGF gene and protein expression (Figure 5) (*p* < 0.05).

### 3.5. Melatonin Regulated HOXA10 Expression through miRNA

**Figure 6:**
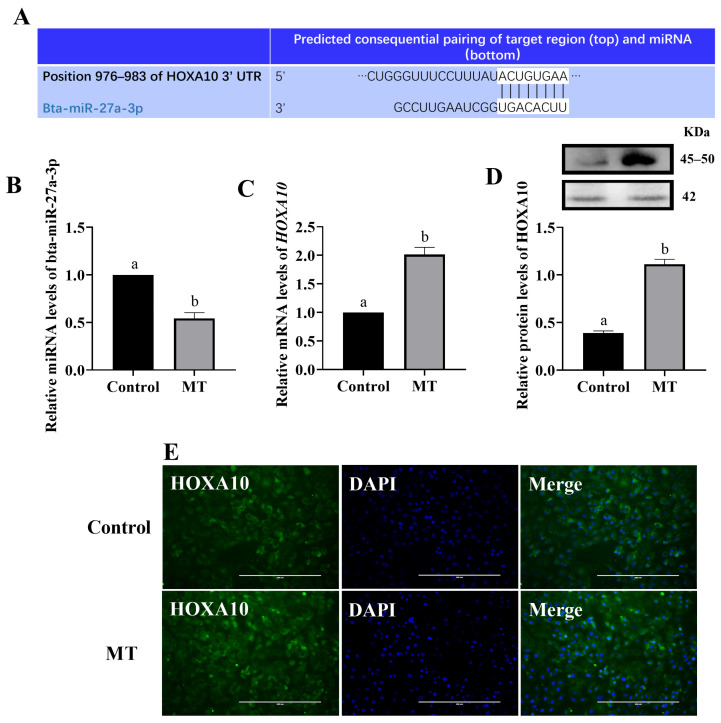
Effect of MT on HOXA10 and bta-miR-27a-3p expression (**A**) predicted binding sites for bta-miR-27a-3p on HOXA10; (**B**) effect of melatonin treatment on the expression of bta-miR-27a-3p in cells; (**C**–**E**) effect of melatonin treatment on the expression of HOXA10 mRNA and protein in cells. Bar = 200 μm. MT: Melatonin. The same letters in this Figure mean insignificant difference (*p* > 0.05), while different letters mean significant difference (*p* < 0.05).

**Figure 7:**
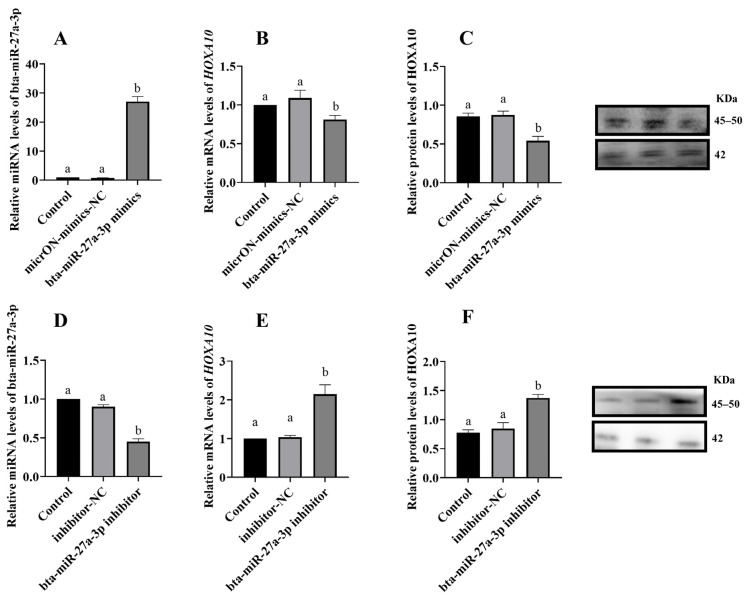
Effect of overexpression and inhibition of bta-miR-27a-3p on the expression of bta-miR-27a-3p and HOXA10 (**A**) the expression of bta-miR-27a-3p when overexpressing bta-miR-27a-3p; (**B**,**C**) the expression of HOXA10 when overexpressing bta-miR-27a-3p; (**D**) the expression of bta-miR-27a-3p during inhibition of bta-miR-27a-3p; (**E**,**F**) the expression of HOXA10 during inhibition of bta-miR-27a-3p. The same letters in this Figure mean insignificant difference (*p* > 0.05), while different letters mean significant difference (*p* < 0.05).

Bioinformatics analysis predicted that miR-27a-3p could bind to 3′ UTR HOXA10. Following MT treatment of the BEEC, there was a significant decrease in miR-27a-3p expression and a significant increase in both the gene and protein expression of HOXA10 (Figure 6) (*p* < 0.05). To further investigate the regulatory relationship between miR-27a-3p and HOXA10, overexpression and inhibition experiments were conducted. The RT-qPCR and Western blot analyses revealed that overexpression of miR-27a-3p significantly increased miR-27a-3p levels and decreased HOXA10 gene and protein expression (*p* < 0.05). Conversely, inhibition of miR-27a-3p significantly reduced miR-27a-3p levels and increased HOXA10 gene and protein expression (Figure 7) (*p* < 0.05).

### 3.6. Regulatory Relationship between SIRT1, AKT, and miRNA

**Figure 8:**
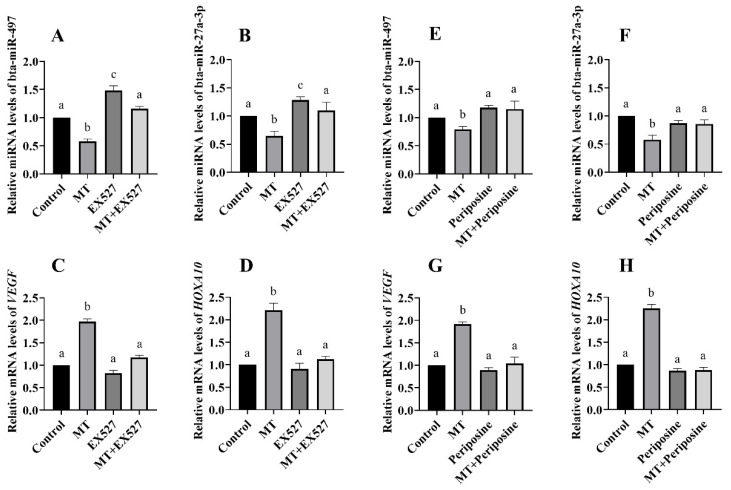
Effects of blocked SIRT1 activation or inhibited AKT phosphorylation on the expression of *VEGF*, *HOXA10*, bta-miR-497, and bta-miR-27a-3p (**A**–**D**) the expression of *VEGF*, *HOXA10*, bta-miR-497, and bta-miR-27a-3p when BEEC were treated with EX527 at a concentration of 1.5 μM; (**E**–**H**) the expression of *VEGF*, *HOXA10*, bta-miR-497, and bta-miR-27a-3p when BEEC were treated with periposine at a concentration of 3 μM. MT: Melatonin.The same letters in this Figure mean insignificant difference (*p* > 0.05), while different letters mean significant difference (*p* < 0.05).

After treatment of the BEEC with the MT and the SIRT1 inhibitor, the RT-qPCR analysis showed that compared to the control group, the expression levels of miR-497 and miR-27a-3p in the MT group were significantly decreased, while the expression levels of *VEGF* and *HOXA10* were significantly increased (*p* < 0.05). In comparison to the MT group, the MT and EX527 co-treatment group showed significantly increased expression levels of miR-497 and miR-27a-3p, along with significantly decreased expression levels of *VEGF* and *HOXA10* (*p* < 0.05). However, there was no significant difference between the MT and EX527 co-treatment group and the control group (Figure 8A–D) (*p* > 0.05). Similarly, after treatment of the BEEC with the MT and an AKT inhibitor, the RT-qPCR analysis demonstrated significant decreases in the expression levels of miR-497 and miR-27a-3p compared to the control group (*p* < 0.05). Meanwhile, the expression levels of *VEGF* and *HOXA10* were significantly increased in the MT group (*p* < 0.05). However, co-treatment of MT and the AKT inhibitor (periposine) led to significant increases in the expression levels of miR-497 and miR-27a-3p compared to the MT group (*p* < 0.05). Additionally, the expressions of *VEGF* and *HOXA10* were significantly decreased in the MT and periposine co-treatment group (*p* < 0.05), with no significant difference observed compared to the control group (Figure 8E–H) (*p* > 0.05).

## 4. Discussion

Early embryo loss is a significant cause of pregnancy failure in dairy cows. The endometrium is the main target organ of embryo implantation, and the success of implantation determines the quality of subsequent pregnancy. Disruptions in endometrial receptivity can lead to early embryonic loss and consequent pregnancy failure [26]. The complexity of the paracrine interactions between the bovine embryo and endometrium, which affect the establishment and maintenance of pregnancy, necessitates further exploration of the underlying mechanisms and regulatory techniques involved in cow implantation [27]. In mice, melatonin has been shown to directly affect embryo implantation by binding to its membrane receptors MTNR1A (MT1) and MTNR1B (MT2), thereby activating several signaling pathways associated with implantation, such as LIF and P53 [28,29]. In our study, melatonin treatment significantly enhanced the proliferation of bovine endometrial epithelial cells. This finding aligns with previous research indicating that melatonin can promote cell proliferation through the activation of the PI3K/AKT and MAPK pathways [30,31]. Our experimental results align with previous findings, demonstrating that melatonin treatment significantly increased the expression levels of PI3K, AKT, and proliferation-related genes cyclinD1 and cyclinE1 in bovine endometrial epithelial cells. This suggests that melatonin promoted BEEC proliferation by activating the PI3K/AKT signaling pathway. Factors influencing endometrial receptivity include endometrial morphology, cytokine expression, vessel number, and hemodynamic markers. During the process of bovine implantation, the number of endometrial blood vessels increased with the number of days, and the expression and regulation of VEGF would affect the vascular remodeling of the implantation [32]. Notably, promoting angiogenesis can enhance endometrial receptivity, with VEGF being a key factor in this process [33]. In our study, melatonin treatment of bovine endometrial cells resulted in significantly higher fluorescence intensity of VEGF protein expression compared to the control group, indicating that melatonin promotes VEGF expression after binding to its membrane receptors. Therefore, melatonin has the potential to stimulate angiogenesis, thereby enhancing the receptivity of endometrial cells in dairy cows. The HOXA10 gene is crucial for regulating endometrial receptivity during the implantation process. In HOXA10-deficient mice, embryos fail to implant into the endometrium, resulting in unsuccessful pregnancies. However, these mice can ovulate normally, and their embryos can survive when transferred to wild-type surrogate mothers [34,35], highlighting HOXA10’s essential role in endometrial receptivity. In our study, the expression level of HOXA10 gene was significantly increased by melatonin treatment of bovine endometrial epithelial cells. These findings, along with previous research, suggest that melatonin improves endometrial receptivity by upregulating key genes such as VEGF and HOXA10, thereby enhancing embryo implantation success rates. Additionally, melatonin has been shown to increase the expression of genes like LIF, HOXA10, and HOXA11 in endometriosis mice. It has also been demonstrated to inhibit VEGF expression via the NF-κB signaling pathway, reducing apoptosis and improving endometrial receptivity in adenomyosis models. These observations support the results of our study [36].

SIRT1, serving as a molecular target for the multifaceted actions of melatonin, has been extensively investigated across various cellular and animal models [37]. Melatonin has demonstrated hepatoprotective effects in mice against alcohol-induced liver injury by inducing SIRT1-mediated signaling pathways [38]. Additionally, it has been shown to retard oocyte aging post-ovulation in mice by activating SIRT1 [39]. Moreover, melatonin modulates oxidative stress in embryos derived from parthenogenetically activated mouse MⅡ oocytes post-cryopreservation and revival through SIRT1 regulation, thus fostering embryonic development [40]. Despite the recognized positive impacts of melatonin on processes such as fertilization, enhancing the quantity of mature oocytes, high-quality embryos, and embryo development, its modulation of uterine endometrial receptivity in cows via SIRT1 remains inadequately explored [41]. In this investigation, melatonin treatment of bovine endometrial epithelial cells significantly upregulated SIRT1 expression, as evidenced by heightened fluorescence intensity following fluorescence staining compared to controls. This suggests that melatonin, upon receptor activation, triggers the expression of the target gene SIRT1. Subsequent treatment with the SIRT1 inhibitor EX527 abolished the melatonin-induced upregulation of PI3K, AKT, HOXA10, and VEGF expression, indicating melatonin’s influence on the PI3K/AKT signaling pathway and receptivity-associated genes HOXA10 and VEGF via SIRT1 modulation. Further validation of this conclusion involved the treatment of bovine endometrial epithelial cells with the AKT phosphorylation inhibitor Periposine. Interestingly, melatonin-induced elevation of SIRT1 expression remained unaffected by Periposine, while melatonin failed to promote VEGF and HOXA10 expression, signifying that melatonin facilitates of VEGF and HOXA10 expression through the SIRT1/PI3K/AKT pathway.

MicroRNA (miRNA) is a small non-coding RNA that plays an important regulatory role during pregnancy, particularly maternal–fetal recognition, and embryo implantation [14,42]. In our study, it was predicted using the TrgetScan8.0 platform that numerous miRNAs target VEGF, including bta-miR-497, bta-miR-16b, bta-miR-29c, and bta-miR-29b, all of which have highly conserved sites. Following melatonin treatment of bovine endometrial epithelial cells, the expression of bta-miR-497 was significantly reduced, while the level of the VEGF gene was significantly increased. Further transfection with a bta-miR-497 mimic and inhibition with a bta-miR-497 inhibitor demonstrated that bta-miR-497 negatively regulates the expression of VEGF. This study is not the first to show that melatonin affects physiological processes by regulating miR-497 expression. Previous research has shown that melatonin alleviates bile acid synthesis induced by alcoholic liver disease by enhancing the expression of miR-497 in mice [43]. Melatonin also attenuated the interstitial transformation of rat glomerular endothelial cells by modulating miR-497 [44]. In addition, we also verified that bta-miR-27a-3p targets the 3′UTR of HOXA10, which was consistent with our previous study [14]. Numerous studies have demonstrated that the expression of the HOXA10 gene can be regulated by miRNAs. For instance, in human endometriosis research, inhibition of HOXA10 expression led to enhanced proliferation, migration, and invasion of hEM15A cells by upregulating miR-27b-3p expression [45]. Additionally, SIRT1 has been shown to regulate miRNA expression. Research indicates that SIRT1 can bind to the miR-20b-3p promoter, mediating the miR-20b-3p/DEPDC1 axis and enhancing L-OHP resistance in colorectal cancer [46]. Similarly in colorectal cancer research, SIRT1 can reduce the transactivation of miR-15b-5p by AP-1 through the deacetylation of activating protein (AP-1) [47]. In this study, melatonin was found to regulate bta-miR-497 and bta-miR-27a-3p in bovine endometrial epithelial cells via the SIRT1/PI3K/AKT pathway, thereby influencing the expression of VEGF and HOXA10. Specifically, bta-miR-27a-3p negatively regulates HOXA10 expression, and bta-miR-497 negatively regulates VEGF expression. However, the precise mechanisms by which bta-miR-497 and bta-miR-27a-3p are regulated through the SIRT1/PI3K/AKT pathway require further investigation.

## 5. Conclusions

Melatonin negatively regulates the expression of bta-miR-497 and bta-27a-3p in bovine endometrial epithelial cells through the SIRT1/PI3K/AKT pathway and positively regulates the expression of VEGF and HOXA10. In this regulatory network, bta-miR-27a-3p negatively modulates HOXA10 expression, while bta-miR-497 negatively modulates VEGF expression.

## Figures and Tables

**Table 1:** Primer sequences for genetic testing.

Gene Name	Serial Number	Primer Sequence (5′→3′)	Product Length (bp)
*β-actin*	NM_173979.3	F: GCGGCATTCACGAAACTACCTT R: TCCTGCTTGCTGATCCACATCT	268
*PI3K*	NM_001206047.2	F: GGATGCTACCTTACGGCTGCTTAG R: CATCTTTGTTGAAGGCTGCTGCTG	87
*AKT*	NM_173986.2	F: AGGACCTGGAGCAGCGTGAG R: GCAGGCAGCGGATGATGAAGG	67
*SIRT1*	NM_001192980.3	F: CAACGGTTTCCATTCGTGTG R: GTTCGAGGATCTGTGCCAAT	138
*HOXA10*	NM_001105017.1	F: TTTCCTTGGCAAAGAGAAGGGCTTG R: GACCTTACACAAACTGGAAGAGAC	99
*VEGF*	M32976	F: CCCAGATGAGATTGAGTTCATTTT R: AGCAAGGCCCACAGGGATTT	245
*LIF*	NM_173931	F: CATCCCTGTCCCAGCAACCTCATG R: CATCCCTGTCCCAGCAACCTCATG	215
*cyclinD1*	NM_001046273.2	F: CTGGTCCTGGTGAACAAACT R: ACAGAGGGCAACGAAGGT	106
*cyclinE1*	NM_001192776.1	F: AAGGAGAGGGATGCGAAGGA R: AATCAGGGAGCAGGGGTTTT	170

*PI3K*: phosphatidylinositol-4,5-bisphosphate 3-kinase, *AKT*: protein kinase B, *SIRT1*: silent information regulator 1, *HOXA10*: homeobox A10, *VEGF*: vascular endothelial growth factor, *LIF*: leukemia inhibitory factor.

**Table 2:** Primer sequence for miRNA quantitative experiment.

Gene Name	Primer Sequence (5′→3′)
U6	F: CTCGCTTCGGCAGCACA R: AACGCTTCACGAATTTGCGT
URP	TGGTGTCGTGGAGTCG
Bta-miR-27a-3p	acactccagctgggTTCACAGTGGCTAA
Bta-miR-497	acactccagctgggCAGCAGCACACTGTGG
Bta-miR-16b	acactccagctgggTAGCAGCACGTAAAT
Bta-miR-29b	acactccagctgggTAGCACCATTTGAAATC
Bta-miR-29c	acactccagctgggTAGCACCATTTGAAAT
Bta-miR-27a-3p (RT-primer)	ctcaactggtgtcgtggagtcggcaattcagttgagCGGAACTT
Bta-miR-497 (RT-primer)	ctcaactggtgtcgtggagtcggcaattcagttgagTACAAACC
Bta-miR-16b (RT-primer)	ctcaactggtgtcgtggagtcggcaattcagttgagGCCAATAT
Bta-miR-29b (RT-primer)	ctcaactggtgtcgtggagtcggcaattcagttgagTTCTCTGT
Bta-miR-29c (RT-primer)	ctcaactggtgtcgtggagtcggcaattcagttgagTAACCGAT

URP: unified reverse primer. RT primers: stem loop reverse transcription primers. The lowercase letter portion of the base sequence in the stem loop RT Primer is a fixed neck loop sequence. The lowercase letters in the real-time fluorescent quantitative primer sequence are protective bases.

**Table 3:** The antibody used in the present study.

Name	Dilution Rate	Manufacturer	Product Number
β-actin rabbit antibody	1:5000	Bioss, Shanghai, China	bs-0061R
SIRT1 rabbit Polyclonal antibody	1:1000	Proteintech, Wuhan, China	13161-1-AP
PI3K mouse monoclonal antibody	1:1000	Proteintech, Wuhan, China	67071-1-Ig
Phospho-AKT(Ser473) mouse Monoclonal antibody	1:5000	Proteintech, Wuhan, China	66444-1-Ig
AKT rabbit polyclonal antibody	1:1000	Affinity, Shanghai, China	AF0908
HOXA10 rabbit Polyclonal antibody	1:1000	Proteintech, Wuhan, China	26497-1-AP
VEGFA rabbit Polyclonal antibody	1:1000	Proteintech, Wuhan, China	19003-1-AP
HRP Goat Anti-Rabbit IgG H&L	1:10,000	Immunoway, China	RS0002
HRP Goat Anti-Mouse IgG H&L	1:10,000	Bioss, Shanghai, China	bs-0296G

**Table 4:** The antibody used in immunofluorescence assay.

Name	Dilution Rate	Manufacturer	Product Number
SIRT1 rabbit Polyclonal antibody	1:200	Proteintech, Wuhan, China	13161-1-AP
Rabbit Anti-HOXA10 Polyclonal Antibody	1:200	Bioss, Shanghai, China	bs-2502R
VEGFA rabbit Polyclonal antibody	1:200	Proteintech, Wuhan, China	19003-1-AP
DAPI	1:500	Biosharp, Heifei, China	BL105A
Goat Anti-rabbit IgG H&L/FITC	1:500	Bioss, Shanghai, China	bs-0295G-FITC

**Table 5:** Primer sequence for overexpression and interference.

Name	Primer Sequence (5′→3′)
NC inhibitor (cel-miR-67-3p)	Antisense strand: UCUACUCUUUCUAGGAGGUUGUGA
NC mimics (cel-miR-67-3p)	Sense strand: UCACAACCUCCUAGAAAGAGUAGA Antisense strand: UCUACUCUUUCUAGGAGGUUGUGA
Bta-miR-27a-3p inhibitor	Antisense strand: CGGAACUUAGCCACUGUGAA
Bta-miR-27a-3p mimics	Sense strand: UUCACAGUGGCUAAGUUCCG Antisense strand: CGGAACUUAGCCACUGUGAA
Bta-miR-497 inhibitor	Antisense strand: UACAAACCACAGUGUGCUGCUG
Bta-miR-497 mimics	Sense strand: CAGCAGCACACUGUGGUUUGUA Antisense strand: UACAAACCACAGUGUGCUGCUG

## Data Availability

None were deposited in an official repository. The data that support the study findings are available upon request.
